# Childhood Leukemia in the Vicinity of the Geesthacht Nuclear Establishments near Hamburg, Germany

**DOI:** 10.1289/ehp.9861

**Published:** 2007-03-15

**Authors:** Wolfgang Hoffmann, Claudia Terschueren, David B. Richardson

**Affiliations:** 1 Institute for Community Medicine, Section Epidemiology of Health Care and Community Health, Ernst-Moritz-Arndt University of Greifswald, Greifswald, Germany; 2 Department of Epidemiology, School of Public Health, University of North Carolina, Chapel Hill, North Carolina, USA

**Keywords:** childhood leukemia, Germany, nuclear installations, standardized incidence ratio, time windows, vicinity

## Abstract

**Background:**

During 1990–1991 a childhood leukemia cluster was observed in the sparsely populated region surrounding two nuclear establishments southeast of Hamburg, Germany. Since then, several new cases have been reported. Recently a possible accidental release of radionuclides in 1986 was hypothesized.

**Objective:**

The objective of this study was to analyze the childhood leukemia incidence in this area since 1990.

**Methods:**

All incident cases (< 15 years of age) were ascertained during 1990–2005 within a 5-km radius of the Krümmel nuclear power plant. We derived standardized incidence ratios (SIRs) using county and national leukemia incidence rates as referents. We stratified analyses by calendar period and attained age, and by subdividing the study region into areas north versus south of the Elbe river.

**Results:**

Fourteen cases were ascertained in the study area, whereas 4.0 were expected based on national referent rates [1990–2005: SIR = 3.5; 95% confidence interval (CI), 1.9–5.9]. The excess was not confined to the early 1990s; for the more recent time period 1999–2005, the SIR is still elevated (SIR = 2.7; 95% CI, 0.9–6.2). SIRs of greatest magnitude were observed for children 0–4 years of age (SIR = 4.9; 95% CI, 2.4–9.0) and for residents south of the Elbe (SIR = 7.5; 95% CI, 2.8–16.4).

**Conclusions:**

The incidence in this region is significantly higher than the childhood leukemia incidence for Germany as a whole. To date, no unique hazards have been identified in this population. The fact that the elevated rates have persisted in this community for > 15 years warrants further investigation.

In the early 1990s, a physician practicing in a community in northern Germany along the Elbe river, southeast of Hamburg, reported an unusual number of childhood leukemia cases among his patients. Between February 1990 and May 1991, five cases of acute leukemia were diagnosed among children living within 5 km of a nuclear power plant and adjacent nuclear research facility. The standardized incidence ratio (SIR) for this 16-month period was 11.8 [95% confidence interval (CI), 4.9–28.3] (Hoffmann et al. 1997).

The German federal states of Schleswig-Holstein, where the nuclear facilities are located, and Lower Saxony, the administrative authority for the Municipality of Elbmarsch, established expert commissions to investigate the cluster (Wassermann et al. 2004; Wichmann and Greiser 2004). Radiation physicists, virologists, meteorologists, and epidemiologists studied the environment (Wassermann et al. 2004). Modestly elevated levels of caesium were detected in rainwater and air samples (Schmitz-Feuerhake et al. 1997), suggesting releases of nuclides above permitted limits; some attic dust samples from houses in the vicinity of the nuclear power plant revealed elevated levels of plutonium and americium (Schmitz-Feuerhake et al. 2003); and some but not all investigations suggested abnormally elevated rates of chromosomal aberrations in peripheral blood lymphocytes of area residents (Brüske-Hohlfeld et al. 2001; Schmitz-Feuerhake et al. 1997).

Schmitz-Feuerhake et al. (2005) postulated that there was an accidental release of radionuclides from the nuclear research facility near this community. The research facility was established in 1958 for the research and development of nuclear reactors for commercial ships. The assertion that there was an unannounced, accidental release of radionuclides in this region (Schmitz-Feuerhake et al. 2005), however, has been an extremely contentious one, leading to dissenting expert opinions from the commissions established to investigate the causes of the regional leukemia excess. A recent publication (Schmitz-Feuerhake et al. 2005) notes that routine environmental sampling in the region provides evidence consistent with an accidental release of radionuclides in September 1986. Support for the theory is buttressed by an expert group of physical chemists that characterized environmental samples of the radionuclides in the region as microspheres containing enriched uranium and thorium, and thus unlikely as fallout, and by the summary statement of the expert committee of Schleswig-Holstein (Wassermann 2004). The expert commission empanelled by the Federal State of Lower Saxony (Wichmann and Greiser 2004), however, concluded that there was no support for the conclusion that the event was an accident. Given the lack of conclusive evidence of an accidental release of radiation, the Northern Germany Leukemia and Lymphoma study derived estimates of doses from routine operations of the nuclear power facility; the resultant dose estimates are orders of magnitude below the level at which any regional excess of childhood leukemia would be expected (Hoffmann et al. 2003). Given our inability to reliably classify residents into subgroups defined by exposure to a suspected accidental release of radionuclides, the objective of this study was to compare the number of cases of childhood leukemia observed in this region with expectations based on county and national childhood leukemia incidence rates over a 16-year period (1990–2005).

## Methods

### Study region

**Figure 1:**
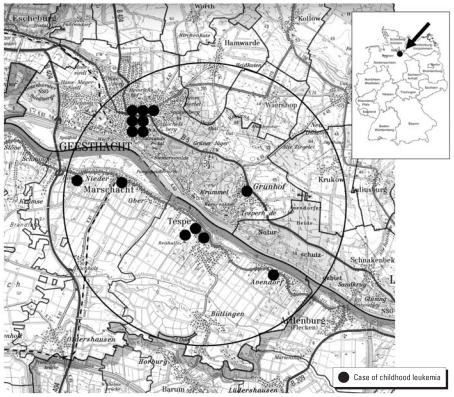
Distribution of childhood leukemia cases (*n* = 14) in the study region 1990–2005 (with national map of Germany inset).

The study region comprises an area defined by a 5-km radius surrounding the nuclear power plant [Kernkraftwerk Krümmel (KKK)] and includes the communities of Marschacht, Tespe, Geesthacht, Hamwarde, Wiershop, and Krukow in the federal states of Schleswig-Holstein and Lower Saxony (Figure 1). The choice of a circular area around KKK reflects the ecologic nature of this study which refers to KKK as a point source. We selected a radius of 5 km for comparability with a previous report (Hoffmann et al. 1997) and with other studies examining disease excesses near posited environmental point sources in Germany (Goans et al. 1997; Hoffmann et al. 1993; Michaelis et al. 1992; Möhner and Stabenow 1993) and in other countries (Laurier and Bard 1999; Shleien et al. 1991). The area of investigation was defined at the start of the study and before the prospective ascertainment of incident cases.

### Case ascertainment

We ascertained all childhood leukemia cases diagnosed among residents of the study region during the period 1990–1998 by review of medical records from specialized treatment centers, hospitals, clinics, and other primary sources in the study region and in Hamburg (Hoffmann et al. 2002). This case ascertainment was conducted as part of the Northern Germany Leukemia and Lymphoma Study, a study of leukemia and lymphoma among children and adults in six northern counties in Germany. Consequently, this case ascertainment procedure provided information on cases among residents of the study region as well as among all residents of the surrounding counties. All cases included in this analysis were documented in the German Childhood Cancer Registry (GCCR; Mainz, Germany). Ascertainment of childhood leukemia cases diagnosed in the study region during the subsequent period, 1999–2005, was based on reports of local physicians and public health authorities. All cases were confirmed by the GCCR. Because of the centralization of pediatric oncology in Germany and the extensive participation in national and international clinical studies, the registry has reached a high degree of medical quality and population-based coverage of all incident cases (Kaatsch and Spix 2005; Kaatsch et al. 1999).

### Population estimates

The Statistics Authorities for the Federal States of Schleswig-Holstein (www.statistik-sh.de) and Lower Saxony (www.nls.niedersachsen.de) provided population estimates for the period 1990–2002 for the communities within the 5-km radius of the KKK, as well as population estimates for the six counties neighboring the study region (data files by calender year). We used the population estimates for 2002 to represent the population counts for 2003–2005, assuming no changes in the population within this period. Population estimates for children < 15 years of age were categorized in four age groups (< 1 year, 1–4 years, 5–9 years, and 10–14 years) to correspond to the tabulations of German national cancer incidence rates.

### Statistical methods

We calculated SIRs as the ratio of the observed numbers of cases to the expected numbers derived by multiplying the age-specific population counts for the study region by German annual childhood leukemia incidence rates for 1990–2005 (Kaatsch and Spix 2005; Kaatsch et al. 1999). Exact 95% CIs were calculated based on methods described by Rothman and Boice (1979).

Childhood leukemia cases and population estimates for 1990–1998 were available for the six counties neighboring the study region. We calculated SIRs for each of the six counties neighboring the study region, deriving the expected numbers of cases by multiplying the age-specific population counts for each county by German annual childhood leukemia incidence rates. We also calculated SIRs for the study region using annual leukemia rates for this six-county region as an alternative to using German national leukemia rates as the referent.

We calculated SIRs with and without stratification by calendar time. Given the small population in the study region, the number of observed and expected cases per calendar year was small. Therefore, rather than calculate annual SIRs, we calculated quinquennial SIRs, summing observed and expected case counts in a series of moving 5-year time windows (i.e., for 1990–1994–1991–1995, … 2001–2004). We also calculated summary SIRs for the period of case ascertainment via the Northern Germany Leukemia and Lymphoma Study (1990–1998) and the period of subsequent follow-up (1999–2005), and examined these period-specific SIRs cross-classified by categories of attained age (0–4, 5–9, and 10–14 years) and with stratification of the communities in the study region into subroups living north versus south of the Elbe river.

## Results

Table 1 reports the characteristics of cases ascertained in the 5-km circular area around the nuclear power plant (KKK). Most (86%) cases were acute lymphatic leukemia, whereas the remainder were acute myeloid leukemia. Most of the cases were males (79%) and were diagnosed at < 5 years of age (71%).

Figure 1 shows the geographic distribution of cases. As suggested by this map, the study area is relatively rural, encompassing several “green” spaces where there are no streets or houses. For reference, 4,667 children < 15 years of age resided in the study region in 1990. Four of the first five cases (and six of the total of 14 cases) lived in villages along the Elbe river on the opposite side of the power plant at the time of diagnosis. Seven cases lived in Geesthacht, the nearest town to the KKK. Only one case’s residence was located northeast of the power plant.

Table 2 shows observed and expected numbers of childhood leukemia cases by calendar year (1990–2005), where expected numbers are based on German national childhood leukemia incidence rates. In 1990 and 1991, five cases of childhood leukemia were observed in the study region, whereas 0.45 cases were expected, consistent with previous reports of an 11-fold excess of childhood leukemia in the region during this period. In total, over the period 1990–2005, 14 cases of acute leukemia were observed in the study area (SIR = 3.5; 95% CI, 1.9–5.9).

The last column of Table 2 shows the expected numbers of cases (for 1990–1998) derived when using childhood leukemia incidence rates for the residents of the six counties surrounding the study region as the referent. The expected numbers of cases in 1990 and 1991 are similar in magnitude to the values derived when using national referent rates, demonstrating that previous reports of excess leukemia in the study region based on comparisons to national referent rates are changed only marginally by use of local childhood leukemia incidence rates as referents. Over the 9-year period 1990–1998, the SIR for childhood leukemia in the study region derived using county referent rates is 3.6 (95% CI, 1.7–6.9), with peak incidence occurring in 1990, 1991, and 1995. In analyses based on national referent rates, the SIR for the period 1990–1998 is 4.2 (95% CI, 1.9–8.1).

The 5-km study region includes parts of two counties, Lauenburg to the north and Harburg to the south. The observed number of cases in Lauenburg during 1990–1998 is very close to the expected number derived when using national referent rates (SIR = 1.02; 95% CI, 0.51–1.83) whereas the observed number of cases in Harburg during the period 1990–1998 is greater than the expected number (SIR = 1.56; 95% CI, 0.96–2.38). Information on childhood leukemia cases was also ascertained for four proximate counties: Luneburg, Pinneberg, Steinburg, and Stormarn. The observed numbers of cases in Luneburg and Stormarn during 1990–1998 were less than expected based on national referent rates (SIR = 0.54; 95% CI, 0.18–1.27; and SIR = 0.95; 95% CI, 0.49–1.66, respectively). The observed numbers of cases in Pinneberg and Steinburg during this period were greater than expected (SIR = 1.22; 95% CI, 0.75–1.89; and SIR = 1.56; 95% CI, 0.85–2.62, respectively).

**Figure 2:**
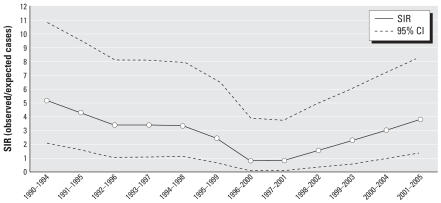
SIRs and associated 95% CIs for childhood leukemia in the study region, calculated in moving 5-year time windows.

Using national referent rates we examined SIRs for the study region during 1999–2005. Over this period, fewer than two cases of childhood cancer were expected in the study region whereas five cases were observed (SIR = 2.7; 95% CI, 0.9–6.2). The average annual expected number of cases during this period was 0.25, suggesting that about one case of childhood leukemia was expected in the 5-km region surrounding the KKK every 4 years. As shown in Table 2, in most but not all calendar periods the observed number of childhood leukemia cases exceeded expectation. We calculated SIRs by moving 5-year time windows to examine the temporal pattern of childhood leukemia incidence in the region via a method that minimizes some of the year-to-year variation in rates (Figure 2). During the first five quinquennial periods of observation (1990–1994–1991–1995, … 1994–1999), the observed number of childhood leukemia cases exceeded the expected number by more than a factor of 3 and the 95% CIs constructed for each of these quinquennial intervals excluded unity. During the next five quinquennial periods (1995–2000–1996–2001, …, 1999–2003), the SIRs ranged between 1.0 and 3.0, and for two quinquennial periods, 1996–2000 and 1997–2001, the SIR was less than unity. The 95% CIs constructed for each of these quinquennial intervals included unity. During the final two quinquennial periods, 2000–2004 and 2001–2005, the SIR increased in magnitude and the 95% CI for SIR calculated for the most recent quinquennial period excluded unity.

Table 3 reports SIRs for childhood leukemia for three age ranges (0–4, 5–9, and 10–14 years). During 1990–1998, SIRs were of greatest magnitude for children 0–4 years of age, intermediate for those 5–9 years of age, and smallest in magnitude for those 10–14 years of age. In 1999–2005, SIRs were also of greatest magnitude for children 0–4 years of age; however, no cases were observed in the age range 5–9 years. Over the total study period, SIRs were above unity for each of the categories of attained age, with the largest magnitude SIR (4.91) observed for the youngest category of attained age at diagnosis.

**Figure 3:**
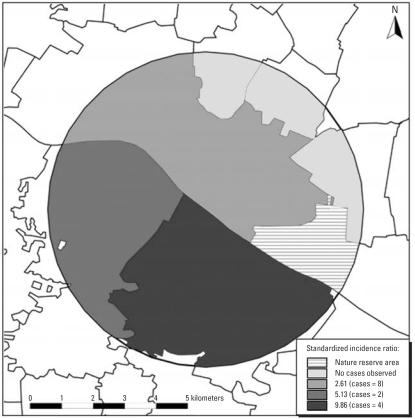
Geographic SIR distribution in 5-km area (1990–2005).

Table 4 reports SIRs for childhood leukemia separately for those communities residing north of the Elbe rive and south of the Elbe river. During 1990–1998, the SIR for childhood leukemia was above unity for residents of the communities north of the Elbe river (SIR = 2.66), but was of most pronounced elevation for the residents in communities south of the Elbe (SIR = 12.68). In 1999–2005, SIRs were elevated for communities north and south of the Elbe river, with approximately 2.5 times the number of cases observed as expected in communities on either side of the river. Over the total study period, the SIR was 2.65 for communities north of the Elbe and 7.65 for communities south of the Elbe. A map of SIRs for 1990–2005 calculated for more detailed geographic units is shown in Figure 3. Eight cases were observed north of the river Elbe. The cases all lived in the largest of four rural communities (3.7 expected). The three rural communities in the northeast had zero cases observed (0.14 expected). Incidence was higher in both rural communities south of the river Elbe (4 cases observed, 0.41 expected; SIR = 9.75).

## Discussion

The incidence of childhood leukemia in the region surrounding the nuclear research and nuclear power facilities in the Elbmarsch municipality is significantly higher than the childhood leukemia incidence rate for Germany as a whole. The excess of childhood leukemia in this region was not confined to the years 1990–1991, when a case cluster was first reported. Most of these are cases of acute lymphoblastic leukemia, conforming to expectations based on German Childhood Cancer Registry incidence data for the period (Haaf et al. 1991).

Several characteristics of this local excess of childhood leukemia warrant comment. The first is the magnitude of the excess: During the period 1990–2005, 14 cases were ascertained in the region defined by a 5-km radius. Compared with several other highly discussed leukemia clusters near European nuclear facilities, this is largest series of childhood leukemia cases reported to date (despite a definition that is relatively narrow in terms of both eligible ages for case inclusion and residential distance from the facility) (Black et al. 1994; Draper et al. 1988; Hoffmann et al. 1997; Viel et al. 1993). For example, the cluster of childhood cancers among children in Seascale, United Kingdom, reported by Gardner et al. (1987) included five deaths from leukemia.

The persistence over time of the excess of childhood leukemia in this region is also noteworthy and bears on consideration of causal explanations for a localized excess of leukemia. Kinlen et al. (1993) have suggested that local excesses of childhood leukemia may occur as a result of an infectious agent that accompanies in-migration to a rural community. Such a hypothesis offers an explanation for an episodic excess of leukemia. However, although some in-migration and population mixing may have occurred in the study region, the fact that the population in this area was fairly stable over the last two decades [see Supplemental Material (http://www.ehponline.org/docs/2007/9861/suppl.pdf)] renders a population-mixing hypothesis less persuasive as an explanation for the persistent elevation of childhood leukemia.

Previous investigators have examined the incidence of childhood cancer in the study region in the decade before 1990. During 1980–1989 the expected occurrence of childhood cancer in the study area was approximately 0.21 cases/year; Schmitz-Feuerhake et al. (2005) reported that two cases of leukemia were observed during 1980–1989, which is slightly less than the number of cases expected based on GCCR incidence data (Schmitz-Feuerhake et al. 2005). An advantage of the current analysis is that it minimizes (for prospectively ascertained cases) potential concerns about post hoc definition of the geographic boundaries of the study region after examination of the spatial distribution cases. Rather, a suspected cluster was identified in the early 1990s near a suspected point source; cases were then ascertained forward in time for the region (periodic case ascertainment, in fact, is still ongoing for this region).

Alternative causal explanations for the elevated childhood leukemia incidence in this region have been suggested, including environmental releases of radionuclides from the nuclear power plant or nuclear research facility located in the study region. In 2004 two expert commissions published reports on the Elbmarsch region. Both commissions emphasized the significantly elevated SIR and the occurrence of additional cases in the area of the Elbmarsch as worrying, but found the results of scientific investigations inconsistent. The commission of Lower Saxony (Wichmann and Greiser 2004) concluded that there is no association between childhood leukemia and the emissions during the normal operations of the nuclear facilities in the Elbmarsch and suggested that not all local risk factors may have been identified and investigated and/or that the people living in this area may carry a certain susceptibility for leukemia. This committee mentioned chance as a plausible alternative explanation. The experts in the Schleswig-Holstein committee concluded that the sudden onset of the cluster and several observed environmental contaminations by uranium, thorium, plutonium, and americium isotopes indicate an accidental release of radioactivity as a likely cause of the elevated SIR for childhood leukemia (Wassermann et al. 2004).

We considered residence (at time of diagnosis) in the study area as part of the definition of our study population. Although we do not have complete residential history information for all cases included in this study, we have confirmed that all cases ascertained between 1990 and 1998 were born in the study region (and resided there until case diagnosis). We do not, however, have residential history information for the most recently diagnosed cases (those diagnosed since 1999).

Studies of exposure biomarkers have also been conducted to investigate the hypothesis that exceptional environmental radiation exposures may have occurred in the region. Of particular interest have been analyses of structural chromosomal aberrations that serve as indicators for radiation-induced DNA damage. Dannheim (1996) assessed chromosomal aberrations in healthy siblings (*n* = 5) of the leukemia cases (Schmitz-Feuerhake et al. 2005) and control children (*n* = 10), reporting an 8-fold increase of “dicentric chromosomes” and “ring chromosomes” in the siblings of the cases. Brüske-Hohlfeld et al. (2001) also used chromosomal aberrations as biomarkers to investigate a potential radiation exposure of random samples of children in the municipality of Elbmarsch (*n* = 42) and a control region (*n* = 30). The authors found no difference in the frequency of dicentric and ring chromosomes between these groups. Schmitz-Feuerhake et al. (1993, 1997) studied chromosomal aberrations in 21 adult cases (19 females and 2 males) living within 5 km of the KKK and 25 controls (9 females and 16 males) who were living in the city of Bremen. The adults from the study region near KKK showed a significant elevation of dicentric chromosomes when compared with the controls (dicentric chromosomes/metaphase: 5-km area 1.77 × 10^−3^; controls 0.46 × 10^−3^; *p*-value < 0.01). Additionally an observed overdispension of dicentric chromosomes in cells of the local residents (Schmitz-Feuerhake et al. 1997) suggested a contribution of densely ionizing alpha emitters. Because of these findings attention has shifted to scenarios presuming an accident with a release of radionuclides as the causing factor. Schmitz-Feuerhake et al. (2005) recently reported supporting evidence for an accidental event in the adjacent nuclear research facility in September 1986. Blood samples were taken from two of the adults again in 1996. Samples of both adults showed elevated rates of dicentric chromosomes in the first measurements in 1992, but the rates had declined back to normal in this follow-up investigation in 1996 (Schmitz-Feuerhake et al. 1997).

A population-based case–control study of leukemia and lymphoma cases diagnosed between 1986 and 1998 (< 75 years of age at time of diagnosis) has been conducted in the six counties around Hamburg (an expanded study region that encompasses approximately 1 million inhabitants). The study was designed to assess exposures to ionizing radiation from routine operations of the region’s nuclear facilities, medical procedures, occupations, pesticide use, and electromagnetic fields. Risk estimates for ionizing radiation for the group of children < 15 years of age with acute lymphatic leukemia did not reveal an explanation of the cluster in the Elbmarsch area (Hoffmann et al. 2002).

## Conclusions

One view of cancer clusters holds that the clustering in space and time of cases routinely occurs by chance (Bellec et al. 2006; McNally et al. 2002; Petridou et al. 1996), although investigations such as the EUROCLUS (Clustering of Childhood Leukaemia in Europe) study suggest that isolated intense clusters of childhood leukemia are rare (Alexander 1998). An alternative view holds that disease causation may be attributed to chance when it is introduced by design in a study (e.g., random exposure assignment); however, in a nonrandomized setting an excess of disease in a place and time necessarily reflects some constellation of causal factors that, in principle, could be identified. To date, there remains substantial uncertainty about the factors that explain the persistently high rate of childhood leukemia in the Elbmarsch region of Germany. More broadly, the evidence of elevated childhood leukemia rates in the region near the KKK in the Elbmarsch region becomes another piece in a growing puzzle constituted by the literature on case–control studies of associations between living in the vicinity of nuclear facilities and childhood leukemia (Gardner 1991; Pobel and Viel 1997). A recent hypothesis of an accident in the nuclear research facility adjacent to the KKK in 1986 (Schmitz-Feuerhake et al. 2005), is challenged because it appears unlikely that such an accident could have escaped environmental surveillance, and no action by public authorities was taken. Further studies of chromosome aberrations might help evaluate the hypothesis of an accidental release of radiation near the KKK, and epidemiologic surveillance should continue to investigate and characterize the evolution of leukemia rates in the region.

## Figures and Tables

**Table 1:** Characteristics of cases of childhood leukemia ascertained in the 5-km study region located in the Elbmarsch, Germany (1990–2005).

Case no.	Diagnosis	Sex	Age at diagnosis (years)	Date of diagnosis (month/year)
1	c-ALL	F	3	Feb/1990
2	c-ALL	M	9	Mar/1990
3	AML	M	9	Apr/1990
4	c-ALL	F	1	Jan/1991
5	c-ALL	M	2	May/1991
6	AML	M	1	Jul/1994
7	T-ALL	M	10	May/1995
8	ALL	M	4	Aug/1995
9	c-ALL	M	3	Jun/1996
10	ALL	F	3	Aug/2001
11	ALL	M	10	Oct/2002
12	ALL	M	4	Mar/2003
13	ALL	M	2	Mar/2004
14	ALL	M	4	Nov/2005

Abbreviations: c-ALL, common acute lymphocytic leukemia; AML, acute myeloid leukemia; F, female; M, male; T-ALL, acute lymphoblastic leukemia of T-cell type.

**Table 2:** Observed and expected numbers of childhood leukemia cases by calendar year in the 5-km study region located in the Elbmarsch, Germany (1990–2005).

Year	No. observed	National referent expected^a^	County referent expected^b^
1990	3	0.22	0.34
1991	2	0.23	0.34
1992	0	0.23	0.22
1993	0	0.24	0.22
1994	1	0.24	0.40
1995	2	0.23	0.21
1996	1	0.24	0.23
1997	0	0.25	0.25
1998	0	0.24	0.28
1999	0	0.27	
2000	0	0.27	
2001	1	0.27	
2002	1	0.27	
2003	1	0.27	
2004	1	0.27	
2005	1	0.27	

a Expected counts were derived using annual age-specific German national childhood leukemia incidence rates as the referent.

b Expected counts were derived using annual age-specific childhood leukemia incidence rates for the six counties surrounding the study region as the referent.

**Table 3:** SIRs for childhood leukemia (< 15 years of age) and observed numbers of cases in two categories of calendar time (1990–1998 and 1999–2005) and three categories of attained age (0–4, 5–9, and 10–14 years).

	1990–1998				1999–2005				Total (1990–2005)			
Age (years)	Obs	Exp	SIR	95% CI	Obs	Exp	SIR	95% CI	Obs	Exp	SIR	95% CI
0–4	6	1.11	5.39	1.98–11.72	4	0.92	4.33	1.18–11.09	10	2.04	4.91	2.35–9.03
5–9	2	0.63	3.20	0.39–11.55	0	0.55	—	—	2	1.17	1.71	0.21–6.16
10–14	1	0.38	2.61	0.07–14.56	1	0.41	2.43	0.06–13.53	2	0.79	2.52	0.30–9.09
Total	9	2.12	4.24	1.94–8.05	5	1.88	2.66	0.86–6.20	14	4.00	3.50	1.91–5.87

Abbreviations: Exp, expected number of cases, derived using German national annual age-specific leukemia incidence rates; Obs, observed number of leukemia cases.

**Table 4:** SIRs for childhood leukemia (< 15 years of age) and observed numbers of cases for communities north versus south of the Elbe river.

	1990–1998				1999–2005				Total (1990–2005)			
Community location relative to the Elbe	Obs	Exp	SIR	95% CI	Obs	Exp	SIR	95% CI	Obs	Exp	SIR	95% CI
North	4	1.71	2.34	0.64–6.00	4	1.50	2.66	0.73–6.82	8	3.21	2.49	1.08–4.91
South	5	0.42	12.04	3.91–28.09	1	0.38	2.63	0.07–14.65	6	0.80	7.54	2.77–16.41
Total	9	2.12	4.24	1.94–8.05	5	1.88	2.66	0.86–6.20	14	4.00	3.50	1.91–5.87

Abbreviations: Exp, expected number of cases, derived using German national annual age-specific leukemia incidence rates; Obs, observed number of leukemia cases.
